# Developmental Robustness: The Haltere Case in *Drosophila*

**DOI:** 10.3389/fcell.2021.713282

**Published:** 2021-07-23

**Authors:** Guillaume Giraud, Rachel Paul, Marilyne Duffraisse, Soumen Khan, L. S. Shashidhara, Samir Merabet

**Affiliations:** ^1^IGFL, ENS Lyon, UMR 5242, Lyon, France; ^2^Indian Institute of Science Education and Research (IISER), Pune, India; ^3^Ashoka University, Sonipat, India

**Keywords:** Hox, transcription, evolution, flight appendage, insects

## Abstract

Developmental processes have to be robust but also flexible enough to respond to genetic and environmental variations. Different mechanisms have been described to explain the apparent antagonistic nature of developmental robustness and plasticity. Here, we present a “self-sufficient” molecular model to explain the development of a particular flight organ that is under the control of the Hox gene *Ultrabithorax* (*Ubx*) in the fruit fly *Drosophila melanogaster*. Our model is based on a candidate RNAi screen and additional genetic analyses that all converge to an autonomous and cofactor-independent mode of action for Ubx. We postulate that this self-sufficient molecular mechanism is possible due to an unusually high expression level of the Hox protein. We propose that high dosage could constitute a so far poorly investigated molecular strategy for allowing Hox proteins to both innovate and stabilize new forms during evolution.

## Introduction

Hox genes are well-known master developmental regulators that have extensively been exploited for diversifying animal body forms during evolution (Pearson et al., 2005; Pick and Heffer, 2012). Numerous cases of morphological diversification have been described as resulting from subtle modulations of the Hox gene expression profile in invertebrates (see for example Averof and Akam, 1995; Stern, 1998; Kittelmann et al., 2018) and vertebrates (see for example Gomez and Pourquié, 2009; Di-Poï et al., 2010; Mallo, 2018). Morphological innovations can also result from changes in the Hox protein sequence itself, as described for abdominal leg repression in arthropods (Galant and Carroll, 2002; Ronshaugen et al., 2002; Pearson et al., 2005; Saadaoui et al., 2011). Thus, despite a fundamental role during embryonic development, which involves a certain degree of stability for the underlying developmental programs, Hox genes remain tolerant for genetic variations and the evolution of phenotypic traits. Here we propose to directly tackle this apparent paradox by focusing on the flight appendage formation in insects in general, and in the fruit fly *Drosophila melanogaster* in particular.

Insects display an astonishing level of morphological diversity, as exemplified in their flight appendages, which differ from one order to the other. Ancestral insects had two pairs of wings on their second (T2, forewing) and third (T3, hindwing) thoracic segments (Carroll et al., 1995), most often of identical or highly similar morphology, as observed in damselflies and dragonflies (Odonata order). Forewings and hindwings can also be of different shape, size and/or color, as observed in the bees (Hymenoptera order) or butterflies (Lepidoptera order). In addition, wings can also be strongly diverged into a new organ, as found in coleopterans, which have developed a protective envelope called elytron in place of the T2 wing, or in dipterans, which have developed a tiny dumbbell-shaped organ called haltere in place of the T3 wing.

What about the role of Hox genes in the morphological diversifications of flight appendages in insects? Most of our current understanding stands from studies in *Drosophila melanogaster* and the beetle *Tribolium castaneum*. Pioneer genetic work in *Drosophila* established the critical role of a single Hox gene, *Ultrabithorax* (*Ubx*), for the repression of anterior wing and the formation of posterior haltere on the third thoracic segment (Morata and Garcia-Bellido, 1976; Lewis, 1978; Bender et al., 1983; Carroll et al., 1995). A similar scenario was observed in *Tribolium*, where *Ubx* was shown to act by repressing the anterior elytron fate for ensuring posterior wing formation in the T3 segment (Tomoyasu et al., 2005).

Another striking feature relates to the relative morphological plasticity of halteres during dipteran evolution despite their critical role of flight. The role of halteres for flight behavior is well established (Dickinson, 1999; Hall et al., 2015): a fly without halteres cannot fly, and these balancing organs produce anti-phase beats and the inertial forces to stabilize the flight. Halteres display a certain level of morphological diversity amongst the different dipteran orders. Not only the shape but also the size (often but not systematically in correlation with the size of the adult insect) of the distal bulb part can fluctuate to some extent. In addition, some variation is observed in the region that connects the distal part of the haltere to the body, in particular in the arrangement of sensory elements (Agrawal et al., 2017). This level of morphological variation underlines that the Ubx-dependent haltere developmental program is not refringent to changes during insect evolution.

The ability of Ubx to specify different flight appendages during insect evolution while also ensuring a robust developmental program presents us with one of the most fascinating yet unsolved paradoxes. Our work aims at tackling this controversial issue, taking the opportunity of this special issue to present a brief research report that supports, together with a previous published work (Paul et al., 2021), a speculative model based on the Hox dosage.

## Results

### From Ubx Expression Level to the Design of an RNAi Screen for Ubx Modulators in the Haltere Disc

**FIGURE 1:**
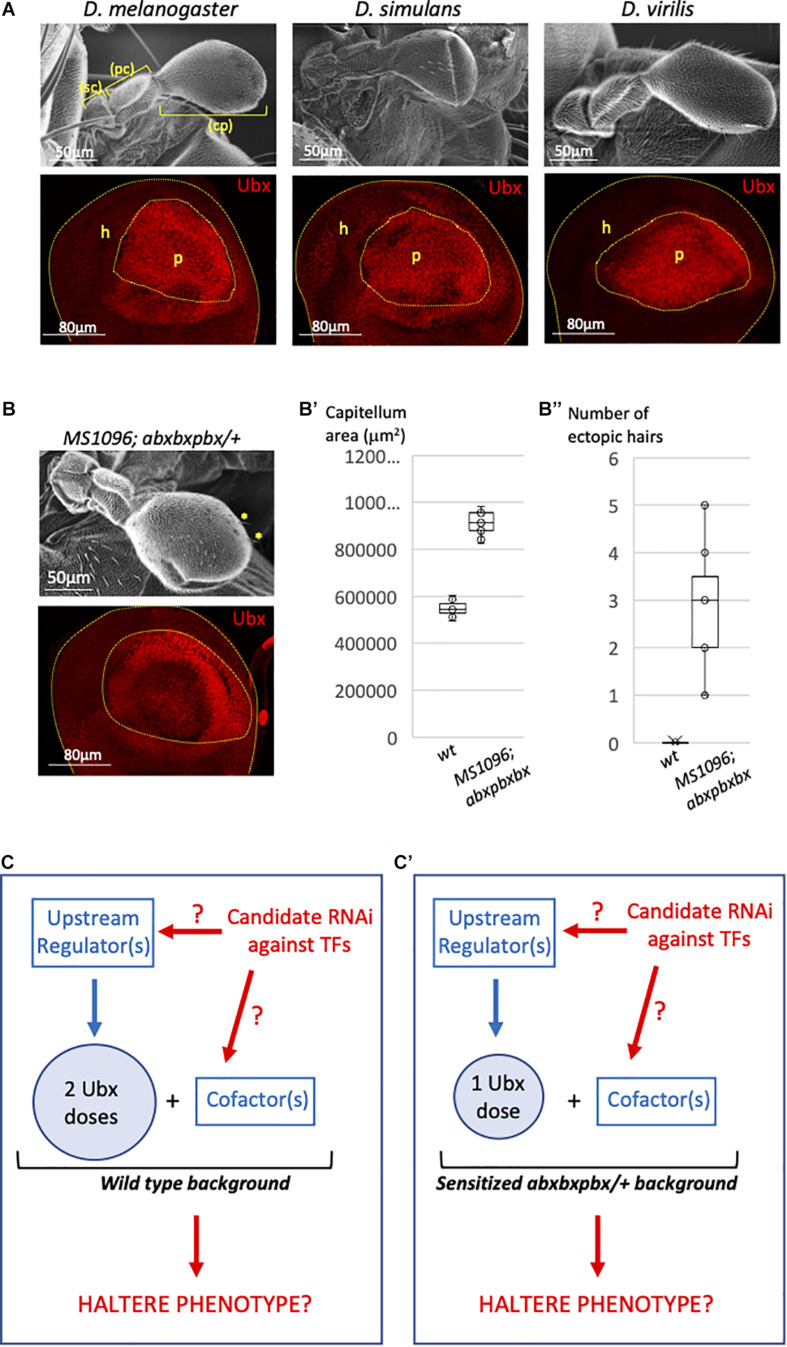
Genetic model for the candidate Ubx regulators screening in the *Drosophila* haltere pouch. **(A)** Ubx (red) is expressed at high level in the pouch of the third instar larval haltere discs of *Drosophila melanogaster*, *Drosophila simulans*, and *Drosophila virilis*. The pouch (p) and hinge (h) regions will give rise to the capitellum (cp) and pedicellum (pc)+ scabellum (sc), respectively. Bottom images are illustrative confocal acquisition. Upper images are illustrative SEM acquisitions of adult halteres. **(B)** Haltere morphology (upper SEM picture) and Ubx expression profile (red, bottom confocal picture) in the haltere pouch in the *abxbxpbx* background context, as indicated. Yellow stars indicated ectopic wing-like bristles **(B’)**. Boxplot representation of the quantification of the adult haltere size in the wild type (*wt*) and *abxbxpbx* backgrounds. **(B”)** Boxplot representation of the quantification of the number of ectopic wing-like bristles in the wild type (*wt*) and *abxbxpbx* backgrounds. **(C–C’)** Model for the candidate RNAi screen that could affect upstream *Ubx* regulators or Ubx cofactors in the *wt* **(C)** or sensitized **(C’)** background. The RNAi screen is targeting transcription factors (TFs).

Ubx is expressed in the entire haltere imaginal disc, but with distinct levels depending on the region: it is highly expressed in the so-called pouch region, which will give rise to the distal bulb called capitellum (Figure 1A), and less strongly expressed in the proximal regions that will give rise to the hinge (composed of the pedicellum and scabellum parts) and metanotum in the adult (Figure 1A; White and Wilcox, 1985; Delker et al., 2019). Early (Irvine et al., 1993), and recent (Delker et al., 2019) work showed that a negative autoregulatory loop contributes to the stabilization of distinct Ubx expression levels along the proximal-distal axis within the haltere imaginal disc of *Drosophila melanogaster*. Considering the robust regulation of Ubx levels within the haltere disc, we asked whether this expression profile was conserved in other *Drosophila* species. We observed the same proximal-distal bias in the third instar larval haltere imaginal discs of *Drosophila virilis* and *Drosophila simulans*, suggesting that the strong expression level of Ubx in the haltere pouch is not trivial (Figure 1A). Accordingly, changes in the Ubx expression level in the pouch have also been shown to increase (upon lower expression levels) or decrease (upon higher expression levels) the size of the capitellum (Crickmore et al., 2009). Interestingly, removing 40% of *Ubx* in a particular genetic background (a heterozygous context for the *abxbxpbx* mutation: Figure 1B (Casares et al., 1996; Paul et al., 2021) led to a significant increase of the size of the capitellum (Figure 1B’) and the apparition of a few wing-like bristles (Figure 1B”). Altogether these observations highlight that high Ubx levels in the pouch allow buffering against changes in the Ubx dose for ensuring a robust development of the haltere bulb. This feature was considered in our attempt to identify additional players that could participate in the Ubx-dependent haltere specification program in *Drosophila melanogaster*.

Our approach relied on a functional candidate RNAi screen that was performed in both the wild type (Figure 1C) and heterologous *abxbxpbx* (Figure 1C′) contexts. Each candidate RNAi was specifically expressed in the pouch with the *MS1096 driver*, with the rationale that they could affect upstream regulators of *Ubx* and/or Ubx cofactors (Figures 1C,C′). In any case, the *abxbxpbx/*+ background was considered as a sensitized context that could allow revealing phenotypes potentially buffered (and therefore not revealed) by the high level of Ubx in the wild type background.

### A Candidate RNAi Screen Reveals Ubx-Autonomous Activity for Haltere Specification

In contrast to the wing, the haltere has never been the object of dedicated genetic screens and a large number of genes is more generally annotated for wing and not haltere development in *Drosophila*.^1^ The master regulatory Ubx protein is known to specify the haltere in part by acting at several hierarchical levels to inhibit the wing developmental program (Akam, 1998; Mohit et al., 2003; Crickmore and Mann, 2006; de Navas et al., 2006a; Pallavi et al., 2006; Hersh et al., 2007; Makhijani et al., 2007; Pavlopoulos and Akam, 2011). Importantly, as mentioned above, ectopic expression of *Ubx* in the wing primordium is sufficient to transform the wing into a haltere, underlining that the wing-transformed tissue contains the set of molecular players that allow haltere development upon the Hox regulatory impulse. We thus decided to perform a candidate RNAi screen by targeting genes described to be expressed in the wing. More particularly, we focused on transcription factors encoding genes that have already been tested in a RNAi screen for wing development (Schertel et al., 2015) and whose expression in the haltere imaginal disc was confirmed by RNA-seq (Khan et al., in preparation; see also section “Materials and Methods”).

**FIGURE 2:**
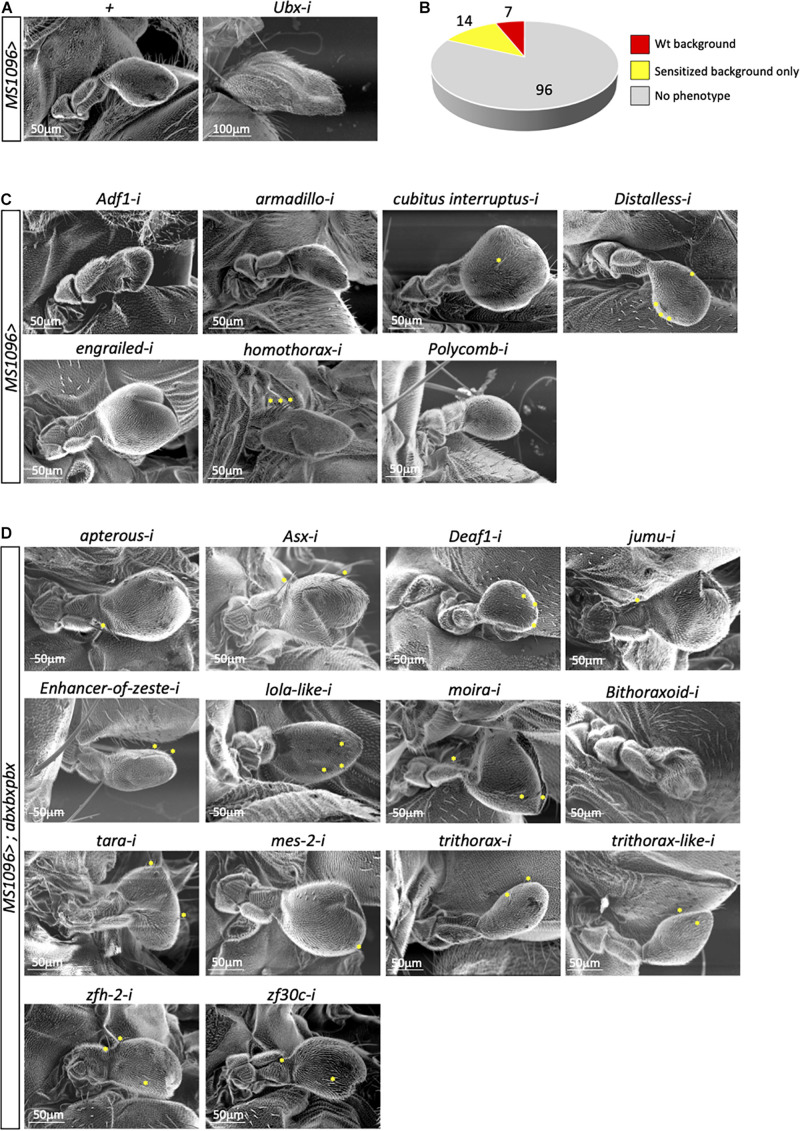
A candidate RNAi screen for transcription factors revealed few potential Ubx cofactors in the haltere disc. **(A)** Control haltere phenotypes in the *MS1096/*+ or *MS1096/*+*; UAS-UbxRNAi/*+ backgrounds, as indicated. **(B)** Summary of the number of genes leading or not to a phenotype (size and/or ectopic wing-like bristles) in the wild type (*wt*, corresponding to MS1096/ +) or sensitized (*abxbxpbx/* +) contexts. **(C)** Illustrative SEM pictures of phenotypes obtained with the different targeted genes in the wild type context, as indicated. **(D)** Illustrative SEM pictures of phenotypes obtained with the different targeted genes in the sensitized context, as indicated. Ectopic wing-like bristles are indicated when present (yellow stars). See also Supplementary Tables 1–3.

In total, we tested 117 genes in the wild type and *abxbxpbx/*+ context backgrounds (Supplementary Table 1). Two different phenotypes of the capitellum linked to a wing-like transformation, therefore to affected Ubx activity, were specifically analyzed: the size and the number of wing-like bristles. As expected, the control experiment with RNAi against *Ubx* led to a strong haltere-to-wing transformation phenotype while the haltere remained unchanged in the *MS1096/*+ background (Figure 2A). In contrast, RNAi against *Antennapedia* (*Antp*), which is expressed in a few cells of the hinge of the haltere disc (Paul et al., 2021), had no effects (Supplementary Table 1). Surprisingly, although the large majority of the 117 tested genes are known to play a role for wing development (Schertel et al., 2015), very few (7/117) affected the haltere capitellum in the wild type (*MS1096/* +) background (Figure 2B and Supplementary Table 2). These genes correspond to different types of TF classes. Two genes, *engrailed* (*en*) and *cubitus interruptus* (*ci*) led to size increase and ectopic bristles (Figure 2C and Supplementary Table 2), highlighting a role for the Hedgehog (Hh) signaling pathway in the *Ubx* developmental program. Interestingly, the regulation of a direct Ubx target gene has been shown to rely on the integration of the Hox and Hh pathways in the haltere disc (Hersh and Carroll, 2005). Two other genes, *Polycomb* (*Pc*) and *Adh transcription factor 1* (*Adf-1*), led to size decrease with (*Pc*) or without (*Adf-1*) ectopic bristles (Figure 2C and Supplementary Table 2). Given the general role of Pc-G proteins as repressors of Hox gene expression (Kassis et al., 2017), we hypothesized that the effect observed with *Pc RNAi* could most likely result from increased *Ubx* expression in the haltere pouch. Finally, RNAi against *armadillo* (*arm*), *Distallless* (*Dll*), and *homothorax* (*hth*) led to ectopic bristles with no significant capitellum size defects (Figure 2C and Supplementary Table 2). Ectopic bristles were specifically observed in the pedicellum in the case of *hth* RNAi, which reflects the expression domain of *MS1096* outside the pouch in the imaginal disc (Paul et al., 2021). Previous work showed that Hth and Ubx are co-expressed in the hinge region and that Hth is downregulating *Ubx* to control its expression level outside the haltere pouch (Delker et al., 2019). The appearance of ectopic bristles on the pedicellum upon *hth* RNAi suggests that Hth could also act as a Ubx cofactor in this region. Accordingly, Ubx and Hth display a striking similarity in their genome-wide binding profiles in the haltere disc (Choo et al., 2011; Slattery et al., 2011) and the two proteins have been shown to interact *in vivo* (Bischof et al., 2018).

The other haltere phenotypes were observed in the *abxbxpbx/*+ background only. However, this sensitized background did not reveal a large number of positive genes (14/117 led to a phenotype: Figure 2D and Supplementary Table 3). Interestingly, 7 genes belong to the Trx group and one to Pc-G (Supplementary Table 3), highlighting that the *abxbxpbx* background is preferentially revealing upstream regulators of *Ubx*. The remaining 6 genes encode for different types of TFs (half of them coding for zinc-fingers containing TFs; Supplementary Table 3). None of these genes had a phenotype on both the size and bristles number upon RNAi, highlighting that their effects were moderate despite the sensitized genetic background.

In conclusion, the candidate RNAi screen revealed few potential cofactors of Ubx in the wild type (Arm, Ci, Dll, En, Hth) or sensitized (Apterous, Beadex, Deaf-1, Jumu, Mes-2, Zfh-2, Zf30c) background. This small number (12/117) was unexpected given the general tendency of Hox proteins to interact with many TFs in *Drosophila* (Baëza et al., 2015; Bischof et al., 2018).

### A Minimal Form of Ubx Is Sufficient to Specify the Haltere Developmental Program in *Drosophila*

**FIGURE 3:**
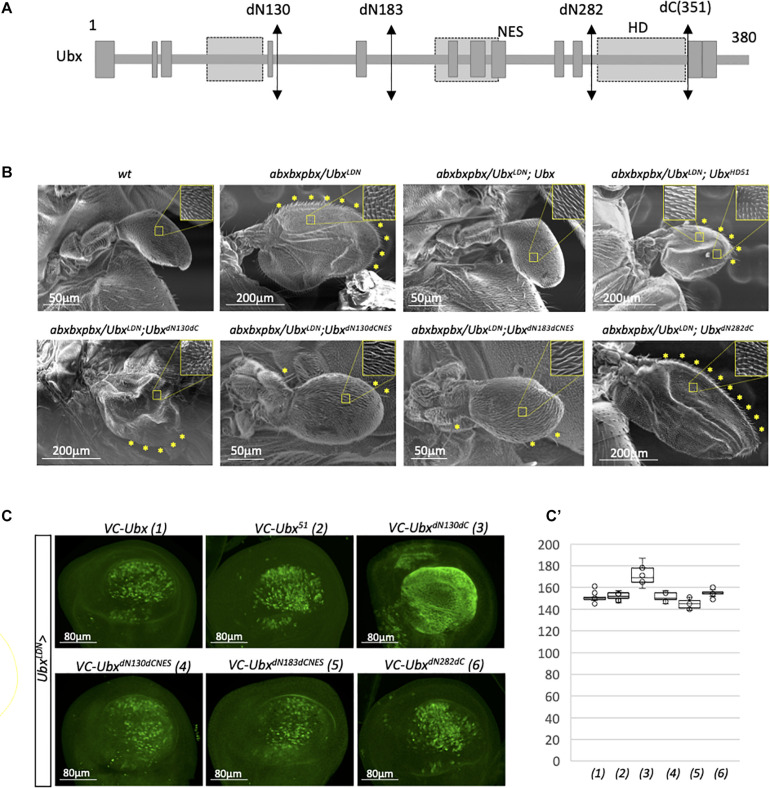
A minimal form of Ubx can rescue the haltere-to-wing mutant phenotype. **(A)** Schematic representation of Ubx with the different sites of deletion. The emplacement of the homeodomain (HD), residue 51 of the HD and unconventional Nuclear Export Signal (NES) are indicated. The NES overlaps with the conserved hexapeptide (HX) motif of Ubx (Duffraisse et al., 2020). **(B)** Illustrative SEM pictures of the activity of the different deleted and mutated forms of Ubx in the rescue assay. The haltere-to-wing mutant context results from the combination of the *abxbxpbx* allele over the hypomorphic *Ubx*^*LDN*^ that corresponds to the insertion of a P*Gal4* in *Ubx* upstream regulatory sequences (Casares et al., 1996). The NES of Ubx has to be mutated for allowing the rescue with the deleted forms. Enlargements depict the haltere-like and/or wing-like hairs in the different genetic backgrounds. Wing-like bristles on the margin are also highlighted (yellow stars). Note that the Ubx^*HD51*^ construct leads to incomplete rescue with the formation of a structure that resembles to a wing in terms of size, hairs (with a mixture of wing-like and haltere-like hairs) and wing-like bristles, but with no obvious veins. In contrast, the Ubx^*dN13*0d*CNES*^ and Ubx^*dN18*3d*CNES*^ constructs lead to an almost complete rescue, although halteres are two times bigger than wild type halteres on average and contain few remaining wing-like bristles. All the phenotypes depicted in the pictures were robustly obtained from two independent experiments for each genetic background. **(C)** Expression of the various mutated and deleted forms of Ubx used in the rescue assay. Immunostaining was performed with an anti-GFP antibody that recognizes the C-terminal fragment of Cerulean (CC) fused to each construct (Duffraisse et al., 2020). The N-terminal deletions induced constitutive nuclear export except when the NES is mutated, as previously described (Duffraisse et al., 2020). **(C’)** Boxplot representation of the quantification of the GFP immunostaining in the haltere pouch upon expression of the different Ubx constructs (1–6) with the *MS1096* driver.

Results obtained from the candidate RNAi screen suggest that Ubx could trigger the haltere developmental program by interacting with an unexpectedly small number of transcriptional partners. In order to explore this molecular aspect further, we dissected the region(s) of the Ubx protein that could be necessary for its activity. The underlying hypothesis was to postulate that a large part of the protein sequence could potentially be dispensable because of a minimum number of interacting cofactors in the haltere disc. The role of the different regions in Ubx was assessed in the context of the genetic rescue of a mutant allelic combination where the haltere is transformed into a small wing in the adult fly (Figure 3A; Casares et al., 1996; Paul et al., 2021). These rescue assays are based on the allelic combination of *abxbxpbx* with an hypomorphic Gal4 insertion (allele *Ubx*^*LDN*^; Casares et al., 1996; de Navas et al., 2006b) that allows to simultaneously express UAS constructs in this background (Brand and Perrimon, 1993). Here, rescue assays were performed with mutated and deleted forms that have previously been used to reveal an atypical nuclear export signal (NES) in Ubx (Figure 3A; Duffraisse et al., 2020).

As expected, the expression of wild type Ubx in the mutant allelic combination led to a complete rescue of the phenotype, with *de novo* formation of a normal haltere (Figure 3B). In contrast, expression of a HD-mutated form that cannot bind DNA (construct Ubx^*HD*51^) led to a partial rescue, with the formation of a structure that displays both wing and haltere characteristics (a similar size to a small wing, with a mixture of wing-like and haltere-like hairs, together with the presence of a number of wing-like bristles on the margin: Figure 3B). This result underlines that the DNA-binding of Ubx is important for its correct activity in the haltere disc. In contrast, DNA-binding integrity was shown to be less critical for *Dll* repression in the epidermis (Sambrani et al., 2013). The first deleted form that we tested was truncated in the N- and C-terminal part (construct Ubx^*dN*130d*C*^) and was not able to rescue the haltere-to-wing transformation phenotype (Figure 3B). This form has previously been shown to be constitutively exported, due to the absence of a NES inhibitory domain in the first 130 amino acids (Duffraisse et al., 2020). We confirmed that Ubx^*dN*130d*C*^ was also constitutively exported in the haltere pouch, explaining that this deleted form was inactive in the rescue assay (Figures 3C,C’). We thus repeated the analysis with the additional mutation of the NES (construct Ubx^*dN*130d*CNES*^; Duffraisse et al., 2020). In this context, the deleted form was able to rescue the mutant phenotype, confirming that the first 130 and last 29 residues of Ubx were not necessary for the haltere developmental program when the protein is properly addressed in the nucleus. An identical level of rescue was observed when deleting even more the N-terminal part in the context of the NES mutation (construct Ubx^*dN*183d*CNES*^; Figure 3B). In contrast, using a minimal form of Ubx that corresponds to the HD only (Ubx^*dN*282d*C*^) was not sufficient for rescuing the phenotype (Figure 3B), although it was correctly localized in the nucleus (Figures 3C,C’). Altogether, these results show that a large part of Ubx is dispensable for its DNA-binding dependent activity in the haltere disc and that the region included between the residues 183 and 351 is sufficient for ensuring the proper haltere developmental program.

## Discussion

The developmental program underlying haltere formation in *Drosophila* is highly robust, which is best exemplified by the fact that almost normal halteres are formed when 40% (*abxbxpbx*/+ background) or 50% (*Ubx*^1^ null allele/+ background) of Ubx level is lost. The wing-like phenotypes (haltere size and ectopic wing-like bristles on the margin) obtained in these genetic backgrounds are weak, which is surprising given that no other Hox gene is expressed in the haltere pouch (there is therefore no redundancy, as it could be in other structures like the leg discs; Wirz et al., 1986; Paul et al., 2021). Strong haltere-to-wing transformation starts to be observed at 60% loss of Ubx (Paul et al., 2021), underlining that the high level of Ubx in the haltere pouch is a way to buffer against mutations that could affect *Ubx* expression. Interestingly, previous work with the *Ubx*^1^ mutant allele also led to the conclusion that haltere development is under strong stabilizing selection (Gibson and Van Helden, 1997) and the notion of “potential variance” with a threshold-dependent response to Ubx haploinsufficency was proposed in this particular developmental context (Gibson et al., 1999). Thus, high Ubx level allows both a stable development of the haltere and subtle morphological changes upon variation. What are the molecular cues underlying Ubx transcriptional activity in this particular context?

Here, we propose that Ubx is working as a “self-sufficient” TF to regulate the set of its target genes and ensure the developmental robustness of the haltere capitellum.

**FIGURE 4:**
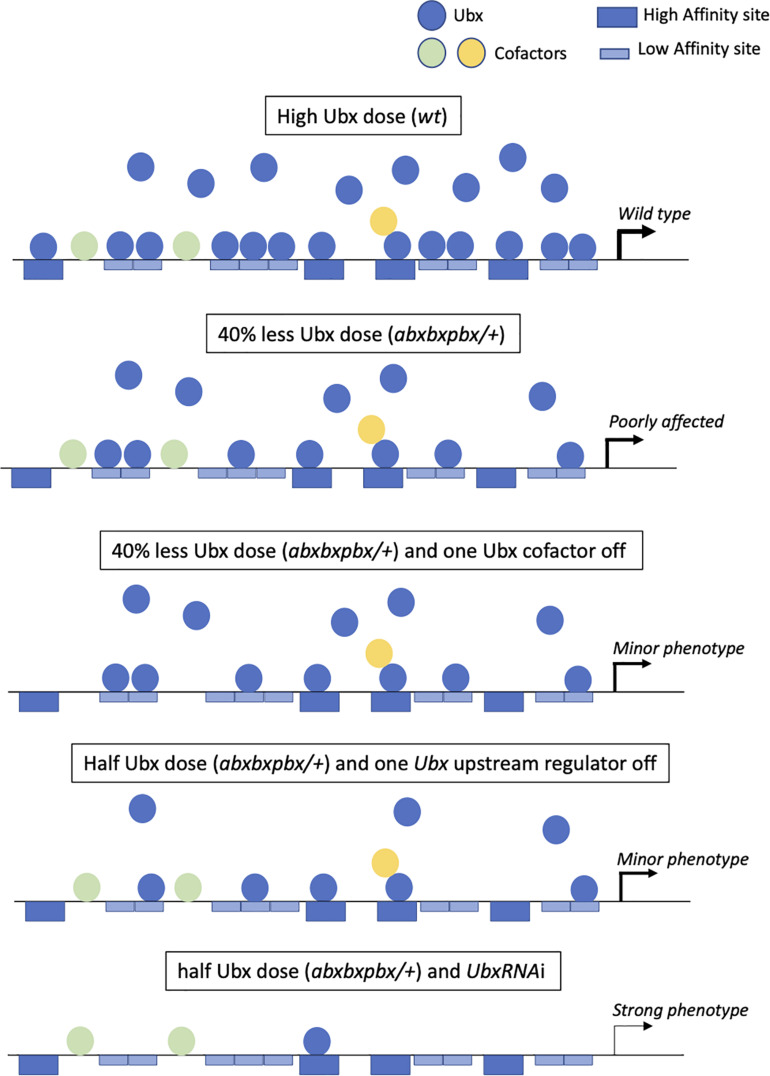
The “self-sufficient” molecular model of Ubx in the haltere disc. Ubx (blue balls) recognizes several redundant high affinity (dark-blue large boxes) and low-affinity (light-blue large boxes) monomeric binding sites in target enhancers at normal (high) doses in the haltere disc. Few cofactors (colored balls) are acting as collaborators (without making dimeric DNA-bound Ubx/cofactor complexes) that modulate the transcriptional output of Ubx. In the context of the heterologous *abxbxpbx* mutant background, there is still enough Ubx molecules to bind on the majority of the redundant monomeric sites, allowing ensuring the haltere developmental program without major morphological variations. The loss of one cofactor in the wild type (not shown in the figure) or mutant context could potentially affect the regulation of some target genes, eventually leading to a subtle phenotype. The haltere morphology could also be moderately affected when targeting an upstream regulator of *Ubx*. Finally, only a strong decrease of Ubx expression level will affect the haltere developmental program, due to the inactivity of most monomeric binding sites in target enhancers.

First, a high dosage of Ubx could be used to recognize several monomeric binding sites on the different target enhancers (Figure 4). For example, Ubx has been shown to repress wing-promoting genes in the haltere disc through the recognition of several consensus monomeric binding sites in repressed target enhancers (Galant et al., 2002; Hersh and Carroll, 2005). Ubx has also been shown to recognize low-affinity binding sites, which are by now established as being critical for ensuring both the specificity and the robustness of Hox-controlled developmental enhancers (Crocker et al., 2015, 2016; Merabet and Mann, 2016). In this context, high doses of the Hox protein could be essential for efficient recognition and binding on these atypical sites *in vivo*. The multiplication of consensus and non-consensus monomeric binding sites could therefore, provide a certain degree of redundancy (Figure 4), making the expression of the enhancer very stable even when half of the monomeric binding sites are not occupied (due to mutations affecting the nucleotide sequence and/or Ubx levels).

Second, a high expression level could confer cofactor-independent activity to Ubx, explaining why our candidate RNAi screen was not successful in revealing phenotypes in the haltere capitellum, even in the sensitized *abxbxpbx* background. Some transcriptional partners might be involved in the case of the regulation of a few target genes and/or be required on a few binding sites in target enhancers, eventually leading to subtle phenotypes that were not captured in the screen. In any case, given that Ubx can perfectly bind to monomeric sites upon high level of expression, we hypothesize that such transcriptional partners could preferentially act as collaborators and modulate Ubx transcriptional activity rather than improving Ubx DNA-binding affinity and/or specificity. The presence of non-consensus binding sites could also be a way to increase monomeric DNA-binding specificity in the absence of cofactors, explaining that only Hox proteins with a similar HD to Ubx (Antennapedia, Antp and Abdominal-A, Abd-A) or strong monomeric DNA-binding activity (Abdominal-B, AbdB) could replace Ubx for haltere specification upon high Ubx-like expression level (Casares et al., 1996; Paul et al., 2021). This self-sufficient molecular model is reinforced by our observation that a minimal form of Ubx can perform the job of haltere specification. This model contrasts with other cofactors’ and collaborator’s based-models (Mann et al., 2009; Merabet and Mann, 2016; Sánchez-Higueras et al., 2019), illustrating the diversity of the molecular strategies that could be used by Hox proteins *in vivo*.

Third, a cofactor-independent mode of activity could allow stabilizing the Ubx developmental program against genetic variation. By definition, requiring fewer cofactors will diminish the number of mutations that could affect/modify the Hox function. Only mutations affecting Ubx levels at various extents could impact on haltere morphology (Figure 4). In this context, the comparison with the wing developmental program is interesting. Indeed, recent work showed that the Hox gene *Antp* is necessary for proper wing formation in *Drosophila* (Paul et al., 2021). However, in contrast to Ubx, Antp is expressed at low levels in specific regions of the pouch of the wing disc. We speculate that this low Hox dose background could serve as a genetic decanalization template for allowing more sensitive phenotypic variability of wings when compared to the haltere capitellum. At the molecular level, the activity of Antp could potentially be more dependent on the interaction with various transcriptional partners when compared to Ubx in the haltere disc. This cofactor-dependent mode of activity could make the wing developmental program more sensitive to genetic perturbations therefore, more plastic for phenotypic variation than the haltere capitellum (Parchem et al., 2007; Soto et al., 2008; Koshikawa, 2020). The same rational could potentially apply in the pedicellum of the haltere, which contains a lower expression level of Ubx when compared to the pouch, for example for varying the arrangement of sensory neurons (Agrawal et al., 2017). Interestingly, one of the rare strong candidate cofactor revealed in our screen (Hth) had a role in this particular region.

Whether a similar dose-dependent scenario could apply in other insect species remains to be investigated. Insect species with similar or dissimilar forewings and hindwings were shown to have similar or dissimilar Hox expression levels in their corresponding wing primordia (Paul et al., 2021). Hox level was also shown to be systematically higher in the hindwing primordia in insect species having different pairs of wings. Whether this differential expression profile is responsible for the phenotypic change is not known. Still, we speculate that a Hox dosage-based model (in contrast to a Hox-specific based model) could constitute a useful molecular mechanism for diversifying flight appendage morphologies in insects. This model is based on the finding that increasing the dose of Antp in the wing disc pouch was sufficient to transform the wing into a haltere (Paul et al., 2021). This phenotype recalls the controversy of flight appendages in Strepsiptera, which regroups endoparasitic insects with an inverted T2 haltere and T3 wing when compared to Diptera (Foottit et al., 2018). Thus, rather than resulting from *de novo* expression of Ubx in T2 as initially proposed (Whiting and Wheeler, 1994), the wing/haltere exchange could “simply” result from an inverted high and low expression level of Antp and Ubx, respectively.

## Materials and Methods

### Drosophila Strains and Genetics Crosses

Drosophila strains were cultured following standard procedures at 25°C. Yellow white was used as a wild-type strain. All the RNAi TRiP lines were obtained from Bloomington (Supplementary Table 1). The following GAL4 were used: *MS1096-Gal4* (Bloomington, #8696) and *Ubx-GAL4^*LDN*^*. UAS RNAi lines were crossed with *MS1096-Gal4* and *MS1096-Gal4; abxbxpbx* followed by incubation at 25°C and emerging flies were observed for the haltere phenotype.

### RNA-Sequencing From Wing and Haltere Imaginal Discs

Wandering third instar larvae from *Drosophila melanogaster* (*CS* strain) were cut and inverted in PBS at 4°C. Wing and haltere imaginal discs were dissected and stored in Trizol separately. RNA extraction and sequencing were performed at Genotypic Technologies at Bangalore, India. Raw reads were filtered for adapter sequences and aligned to the dm6 genome using the HISAT2 software. The full sequencing data will be available in another work (Khan et al., in preparation).

### Immunofluorescence Assay in *Drosophila* Imaginal Discs

Imaginal discs were fixed following dissection in 4% paraformaldehyde (methanol free) for 15 min. Washes were done with 1 × PBS 0.1%TritonX solution (PBTx). Samples were then blocked with 2% BSA solution for 2 h. Primary antibodies were incubated for ON at 4°C and then washed in PBTx and secondary antibodies incubated for 2 h at room temperature. Samples were then washed in PBTx and mounted in Vectashield (Vector laboratories) for confocal acquisition. Primary antibodies used were mouse anti-Ubx/ABD-A FP6.87 (1:20; DSHB) and rabbit anti-GFP PABG1 (1:500; Chromotek).

### Imaging

The adult Drosophila appendage phenotype images were taken by Scanning electron microscope Hirox SH-3000. All the fluorescence microscopy images of haltere imaginal discs were captured using confocal Zeiss LSM 780. Images were captured at a 1,024 × 1,024 pixel resolution using 40x oil objective. The expression levels were quantified by measuring the intensity of GFP using the histogram function of the FIJI Software. The threshold was subjected to minute adjustment (using the « Image calculator » function) to create an image containing all positive nuclei (using the « Subtract » function) that were analyzed for fluorescence quantification (using the « analyze particles » function) and deduce the mean fluorescence intensity.

## Data Availability Statement

The original contributions presented in the study are included in the article/Supplementary Material, further inquiries can be directed to the corresponding author/s.

## Author Contributions

GG: execution of the experiments, data analysis, and formal analysis. RP: conceptualization, execution of the experiments, data analysis, formal analysis, and writing. MD: execution of the experiments. SK and LS: conceptualization and writing. SM: conceptualization, data analysis, formal analysis, and writing. All authors contributed to the article and approved the submitted version.

## Conflict of Interest

The authors declare that the research was conducted in the absence of any commercial or financial relationships that could be construed as a potential conflict of interest.

## Publisher’s Note

All claims expressed in this article are solely those of the authors and do not necessarily represent those of their affiliated organizations, or those of the publisher, the editors and the reviewers. Any product that may be evaluated in this article, or claim that may be made by its manufacturer, is not guaranteed or endorsed by the publisher.

## Footnotes

1 https://flybase.org/

## Supplementary Material

Supplementary Table 1 Full list of the candidate genes tested in the RNAi screen. The type of DNA-binding domain (DBD) and Bloomington RNAi line number are indicated. Boxes highlighted in yellow and orange denote genes that affect haltere formation in the wild type or sensitized background, respectively. Expression levels of each tested candidate gene analyzed from RNA-seq data are given in the standard RPKM (reads per kilobase per million) unit in the wing and haltere discs.

Supplementary Table 2 List of genes leading to haltere phenotype upon RNAi in the wild type context. Haltere size and ectopic bristle quantifications are indicated. These phenotypes were reproducibly obtained from two independent experiments.

Supplementary Table 3 List of genes leading to haltere phenotype upon RNAi in the sensitized context. Haltere size and ectopic bristle quantifications are indicated. These phenotypes were reproducibly obtained from two independent experiments.
